# The use of different reference foods in determining the glycemic index of starchy and non-starchy test foods

**DOI:** 10.1186/1475-2891-13-50

**Published:** 2014-05-31

**Authors:** Bernard J Venn, Minako Kataoka, Jim Mann

**Affiliations:** 1 Department of Human Nutrition, University of Otago, PO Box 56, Dunedin New Zealand

## Abstract

**Background:**

Glycemic index (GI) is intended to be a property of food but some reports are suggestive that GI is influenced by participant characteristics when glucose is used as a reference.

**Objective:**

To examine the influence of different reference foods on observed GI.

**Design:**

The GIs of five varieties of rice and a sugary beverage (LoGiCane™) were tested in 31 European and 32 Chinese participants using glucose or jasmine rice as reference foods. The GIs of two ready-to-eat breakfast cereals (Kellogg’s cornflakes and Sustain) were tested in 20 younger and 60 older people using glucose or Sustain as reference foods.

**Results:**

The GIs of rice tended to be higher in the Chinese compared with the Europeans when glucose was used as a reference (jasmine 80 vs 68, P = 0.033; basmati 67 vs 57, P = 0.170; brown 78 vs 65, P = 0.054; Doongara 67 vs 55, P = 0.045; parboiled 72 vs 57, P = 0.011). There were no between-group differences in GI when jasmine rice was the reference. The GIs of breakfast cereals tended to be lower in younger compared with older groups (cornflakes 64 vs 81, P = 0.008; Sustain 56 vs 66, P = 0.054). There was no between-group difference in the GI of cornflakes when Sustain was the reference (cornflakes 115 vs 120, P = 0.64). There was no ethnic difference in GI when glucose was the reference for another sugary food (LoGiCane™ 60 vs 62; P = 0.69).

**Conclusions:**

A starchy reference may be more appropriate than a glucose beverage when attempting to derive universally applicable GI values of starchy foods.

**Trial registration:**

The Chinese/European trial is registered with the Australian New Zealand Clinical Trials Registry as ACTRN12612000519853.

## Introduction

The suggestion that the blood glucose raising potential of food might be an appropriate means of making carbohydrate recommendations for people with diabetes mellitus was first suggested by Otto and colleagues nearly 40 years ago [1]. Subsequent studies in which postprandial glycemia was measured were undertaken among a variety of population groups comprising people with diabetes or those with normal glucose tolerance [2,3]. Due to quantitative differences in postprandial blood glucose excursions between people with and without diabetes and a lack of standardization of testing, comparisons among studies were generally not possible and the approach was not widely used for developing dietary recommendations.

However, in the early 1980s the concept of the glycemic index (GI) was introduced in which the incremental postprandial blood glucose area under the curve (iAUC) resulting from the ingestion of a test food was expressed as a percentage of the iAUC of a ‘reference’ or ‘standard’ glucose beverage containing an equivalent amount of available carbohydrate [4]. With each person acting as his or her own control, it was reasoned that the GI of a food (the ratio of postprandial iAUCs) applies to all people regardless of their glucose tolerance status [5] and that the concept could provide a useful approach for determining the suitability of carbohydrate containing food for people with diabetes.

Later, some investigators argued that white bread was physiologically a more relevant standard than a glucose beverage when comparing the glycemic response to carbohydrate containing food [6]. To enable comparisons to be made between GIs calculated using different reference foods, a glucose to white bread conversion factor was derived [5]. The use of different standards was acknowledged as acceptable practice in a Food and Agriculture Organization/World Health Organization Nutrition paper in which it was concluded that the standard food could be white bread, a glucose beverage, or indeed another food providing that a relationship had been established between the alternative standard and either white bread or glucose [7].

It is unclear how the choice of reference food impacts on the underlying principle that GI represents a property of the food. Indeed, ethnic differences in GI have been reported when using glucose beverage as the reference [8]. Using relatively large samples we report here on between-group comparisons in the GI of several foods using either glucose or an alternative starchy reference food.

## Methods

Data are derived from two studies. One study involved the GI determination of rice and sucrose in Chinese and European groups using both a glucose beverage and jasmine rice as reference foods; in the other study, the GI of a breakfast cereal was tested in younger and older participants using a glucose beverage and a breakfast cereal as a reference food.

In the Chinese/European study, the participants were 32 self-identified Chinese and 31 European volunteers aged between 18–50 y [9]. These participants tested a glucose beverage (Carbotest® 50 g glucose drink, Lomb Scientific, Australia) and jasmine rice (twice); and sucrose (LoGiCane™, Horizon Science, Australia), brown, Doongara and basmati rice (SunRice, Ricegrowers Ltd., Australia), and parboiled rice (Uncle Ben’s, MasterFoods Australia New Zealand) once. The rice was cooked in a rice cooker using the same rice to water ratio throughout the study (dependent upon rice variety) and served in portions containing 50 g available carbohydrate. A commercial laboratory (AsureQuality Ltd, Auckland New Zealand) used an AOAC method to test the amount of available carbohydrate in each rice variety [10]. To prepare a test sample of the sugar LoGiCane™, 50 ± 0.5 g was weighed using a scientific scale accurate to 0.01 g (Sartorius, USA). The sugar was dissolved with a small amount of hot water then topped up to 300 mL with carbonated water (kiwi® blue, Coca Cola-Amatil Ltd.). The sucrose beverage was stored in a refrigerator overnight before the test day.

The other study involved a comparison of breakfast cereals between a group of 20 people aged 19–32 y and 60 people aged 56–86 y. The younger and older groups tested a glucose beverage three times and twice, respectively, whilst both groups tested two ready-to-eat breakfast cereals, Kellogg’s® Cornflakes and Sustain. The glucose beverage and cereals contained 50 g available carbohydrate; the cereals were tested for starch content (Boehringer Mannheim kit, Germany) and sugars by chromatography [11].

The presence of chronic disease, use of medications influencing glucose metabolism, food allergies and pregnancy excluded participation. Based on standard deviations obtained from previous work in our laboratory, two samples of 30 people had 80% power to detect a GI difference of 10 units using an alpha of 5%. Participants attended the testing facility on each occasion after a 10 h overnight fast. Fasting capillary blood samples were collected before food consumption and postprandially at 15, 30, 45, 60, 90, and 120 min. Food was consumed at an even pace over 15 minutes and participants remained seated throughout the test period. Blood glucose was measured using Hemocue Glucose 201 Analyzers (HemoCue AB, Ängelholm, Sweden).

Incremental area under the blood glucose curve (iAUC) was calculated using the trapezoidal method and ignoring the area below baseline [7]. The iAUCs were log transformed and an individual’s GI calculated by expressing the iAUC of the test food relative to the mean iAUC of the reference food (Glucose or Jasmine rice). The individual GI values were averaged within a group to represent the group mean GI with 95% confidence intervals. Factors to convert GI_(glucose scale)_ to GI_(Jasmine scale)_; and GI_(glucose scale)_ to GI_(Sustain scale)_; were determined by calculating the appropriate conversion factor for each individual and averaging within a group to represent the group mean conversion factor. A mixed model accounting for correlations among the measures, including an interaction term between food and group, was used to test for difference between the groups for AUC and GI. The characteristics of the samples were compared using t-tests for continuous variables ((age, body mass index (BMI), fasting glucose)) and chi-squared tests for categorical variables (sex).

The Human Ethics Committee of the University of Otago approved both studies.

## Results

The mean (SD) age and body mass index (BMI) of the European group was 34.3 (8.18) y and 25.8 (4.77) kg/m^2^, the Chinese were aged 33.4 (8.44) y with a BMI of 22.9 (2.74) kg/m^2^; the BMIs of the groups were different (P < 0.05). There was no between group difference in fasting glucose with the Chinese and European groups both having a mean of 4.8 and a standard deviation of ~0.4 mmol/L. There was no difference in sex distribution between groups (P = 0.71).

**Figure 1:**
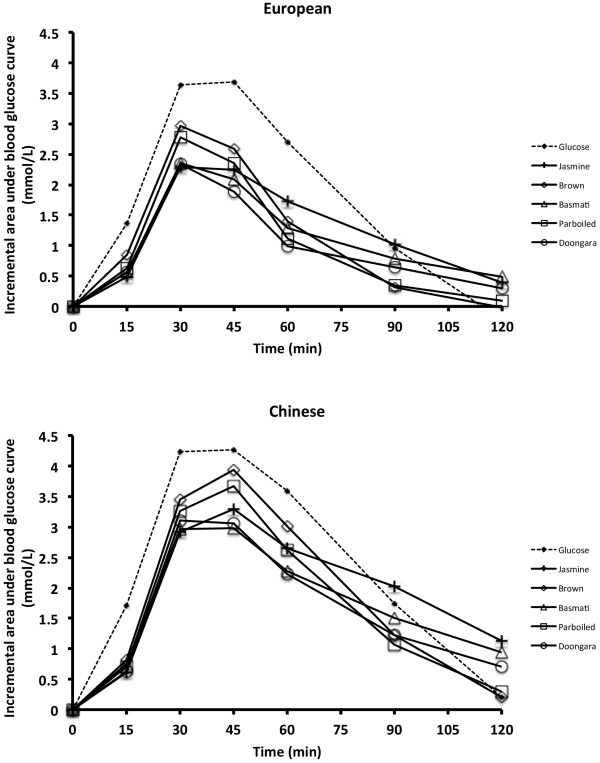
**Mean incremental blood glucose area-under-the-curve (iAUC) for 31 European and 32 Chinese consuming a glucose beverage and five varieties of rice.** Note the rice curves are closer to the glucose curve for the Chinese than for the Europeans, reflecting differences in glycemic index between the groups.

The postprandial iAUCs were higher in the Chinese compared with the European; and higher in the older compared with the younger groups, for all foods tested (Table 1). When using a glucose beverage as the reference, there was no between-group difference in the GI of sucrose, however, the GI of jasmine, Doongara and parboiled rice were significantly higher for the Chinese compared with the European group by 12 to 15 GI units (Table 2). The tendency for higher GI in the Chinese (10 to 13 GI units) was also apparent for basmati and brown rice, although this did not reach statistical significance. On the other hand, when jasmine rice was used as reference food, there were no significant differences in GI for any of the rice varieties with between-group values occurring within 2 to 6 GI units. The mean (95% CI) factor for converting from the Jasmine rice scale to the glucose scale was 0.8 (0.72, 0.90) for the Chinese group and 0.7 (0.61, 0.76) for the European group, and the conversion factors were significantly different from each other (p = 0.038). Plots of the postprandial glucose concentrations are given in Figure 1. There is a larger separation between the glucose beverage and the rice varieties for the European group than for the Chinese group, reflecting the ethnically different GIs given in Table 2.

**Table 1:** Mean (95% CI) incremental area-under-the-curve (iAUC) blood glucose concentrations (mmol/L•min) comparing European vs Chinese groups and younger vs older groups

	**European (n = 31)**	**Chinese (n = 32)**	**P**
Glucose	201 (169, 235)	274 (234, 311)	0.005
Jasmine	140 (117, 171)	225 (187, 253)	< 0.001
Basmati	116 (85, 153)	184 (144, 215)	0.001
Brown	129 (109, 151)	210 (178, 250)	< 0.001
Doongara	109 (94, 127)	179 (150, 215)	< 0.001
Parboiled	112 (95, 136)	194 (165, 227)	< 0.001
Sucrose	120 (99, 146)	169 (142, 203)	0.013
	Younger (n = 20)	Older (n = 60)	P
Glucose	174 (151, 197)	274 (248, 303)	< 0.001
Cornflakes	109 (81, 148)	220 (195, 247)	< 0.001
Sustain	95 (80, 114)	182 (158, 203)	< 0.001

**Table 2:** Mean (95% CI) Glycemic Index (GI) values determined using different reference foods in European (n = 31) and Chinese (n = 32) groups

**Food**	**GI**_**(glucose reference)**_			**GI**_**(jasmine reference)**_		
	**European**	**Chinese**	**P**	**European**	**Chinese**	**P**
Jasmine	68 (61, 76)	80 (72, 90)	0.03	NA	NA	NA
Basmati	57 (49, 67)	67 (58, 77)	0.17	84 (72, 99)	83 (72, 95)	0.88
Brown	65 (57, 74)	78 (68, 89)	0.05	95 (84, 108)	97 (85, 110)	0.85
Doongara	55 (48, 63)	67 (58, 76)	0.04	81 (71, 92)	83 (73, 94)	0.79
Parboiled	57 (50, 64)	72 (63, 82)	0.01	83 (73, 94)	89 (78, 102)	0.42
Sucrose	60 (54, 67)	62 (55, 70)	0.69	88 (77, 101)	78 (68, 89)	0.19

*NA* Not Applicable.

In the comparison of age groups, the BMI of the younger group (23.4 (2.6) kg/m^2^) was lower than that of the older group (27.1 (4.6) kg/m^2^). Fasting plasma glucose was higher in the older compared with the younger group, 4.9 (0.6) and 4.5 (0.4) mmol/L (P < 0.001), respectively. There was no difference in sex distribution between groups (P = 0.31). All of the older group and 95% of the younger group were of European descent. When using the glucose beverage reference, the GI of cornflakes was higher in the older compared with the younger group by 17 GI units (P = 0.008) with a tendency for a higher GI (10 GI units) for Sustain (P = 0.054) (Table 3). When using Sustain as the reference food, there was no between-group difference in the GI of cornflakes (P = 0.64).

**Table 3:** Glycemic Index (GI) values determined using different reference foods in younger (n = 20) and older (n = 60) groups

**Cereal**	**GI**_**(glucose reference)**_			**GI**_**(Sustain reference)**_		
	**Younger**	**Older**	**P**	**Younger**	**Older**	**P**
Cornflakes	64 (56, 74)	81 (74, 88)	0.01	115 (91, 144)	120 (110, 133)	0.64
Sustain	56 (49, 65)	66 (61, 72)	0.05	NA	NA	NA

*NA* Not Applicable.

## Discussion

Different GI values for different groups were found when a glucose beverage was used as the reference when testing starchy foods. These findings are contrary to the basic concept that the index represents a property of the food independent of the consumer [5]. Indeed, it has been reported that GI was not different among groups comprising people with impaired glucose tolerance; diabetes mellitus; lean with normal glucose tolerance; and obese with normal glucose tolerance [12]. Thus, although the BMI of our Chinese and European groups differed, and the BMI of our younger and older participants differed, this should not have affected the GI. We confirmed this using the Chinese and European data in a regression analysis whilst adjusting for BMI in which the statistical significance and the inference were unchanged (data not shown). Additionally, if BMI was postulated to affect GI, a higher BMI was associated with a lower GI in the Europeans whilst in the elderly, the opposite was the case; a higher BMI was associated with a higher GI. Thus, we consider it unlikely that BMI provides an explanation for the observed differences in group GI.

In the series of tests presented here, GI differences for the same food differed by as much as 10 – 17 GI units when considering different ethnic groups or age categories. Such a difference is substantial given that there is only 15 GI unit difference between the nominal ‘high’ (≥70) and ‘low’ (≤55) GI classification. The extent of the group differences even affected the classification. For example, cornflakes was medium GI in the younger group and high GI in the older group. For the European group, the varieties of rice we tested would generally be classified as medium GI with the confidence intervals in the low to medium GI range whereas in the Chinese, rice would be classified as a medium to high GI food. Wolever and colleagues, using a glucose reference, reported a similar finding in which white bread was medium GI in a group of Caucasians (GI = 66) and high GI in non-Caucasians (GI = 78) [8]. Identifying a problem with the reference food suggest that changes should be made to the testing methodology of starchy foods to remove this source of variability, thereby enabling the consistent categorization of GI, necessary both for dietary advice and for generalizability on food labels.

Different GIs for the same food are also found in the International Tables of GI [13]. For example, under ‘boiled white Basmati rice’ the GI values range from 43 to 69. Some of this variability may be due to the growing, processing and cooking methods as alluded to in the preface of the International Tables. However, despite standardization by supplying a single source of long grain white rice with cooking instructions to seven GI testing centres around the world, the GI of the rice was still variable ranging from 55 to 87 [14]. It is of note that age and ethnic composition of the groups differed among the centres although it was stated that these had no significant effect on GI. Some of the variability could have been due to laboratory differences in GI testing protocol. In our series of experiments in which rice was tested in Chinese and European groups, the source of rice, cooking and laboratory methods were all standardised. Similarly, in the young and older participants, the breakfast cereals and testing procedures were the same for both groups. Hence, the observed differences in GI when using a glucose beverage as a reference food are attributable to differences in group characteristics.

An effect of group characteristics on GI was apparent when people were dichotomized as having high or low salivary alpha-amylase activity [15]. A significant between-group difference in postprandial glucose response was noted after participants had consumed a corn starch solution but there was no between-group difference in response to the glucose reference beverage. From an earlier observation, Wolever and colleagues had proposed that the use of a glucose beverage may lead to false conclusions regarding the relevance of observed ethnic differences in GI if starch digestion differed by ethnicity [8]. We measured salivary alpha-amylase concentrations in our Chinese and European groups and found these did not explain the different GIs when glucose was used as a reference [9]. However, the majority of starch digestion occurs in the small intestine via the action of pancreatic alpha-amylase [16] with heterogeneity noted in the entire amylase gene family [17]. Although variability in alpha-amylase could plausibly account for differing rates of starch digestion between Caucasians and non-Caucasians, and between groups having different salivary amylase characteristics, we have found no reports suggesting that alpha-amylase is raised in older people. One possibility is that the elderly are more prone to hypochlorhydria and achlorhydria [18]. The activity of salivary amylase is pH dependent [19] such that a deficiency or lack of stomach acid may enable salivary alpha-amylase to continue working whilst the food is in the stomach.

The potential for different rates of starch digestion are indicative that a solid starchy food may be a physiologically more relevant reference than a glucose beverage when testing the GI of starchy foods. Rice has been used as a reference in a Japanese study due to availability and palatability [20] and although white bread used to be preferred [6], glucose was subsequently recommended as the reference [14]. Consequently, in the International Tables of GI, the GI values of many starchy foods have been determined with reference to glucose [13]. How generalizable those values are is unclear, with our work and that of others presented here being suggestive that for GI to have a universal value, regardless of ethnicity, age or other participant characteristics, that the choice of reference is important. A simple conversion from one reference scale to the other using a multiplicative constant does not resolve the problem. The factor we obtained for converting from a Jasmine rice scale to a glucose scale was 0.8 for the Chinese participants. This is the same value as that found by Sugiyama and colleagues converting from a rice scale to a glucose scale in a Japanese sample [20]. However, the conversion factor for Jasmine rice to glucose in our European sample was 0.7; and this was significantly different to the conversion factor for the Chinese.

For sugary foods, the use of glucose as a reference may be appropriate. In a study by Wolever and colleagues, there were no ethnic differences in GI for chocolate chip cookies and fruit bars, notably with the majority of carbohydrate in those foods coming from sugars rather than starch [8]. Similarly, for the sugary food we tested (LoGiCane™), there was no ethnic difference in GI, supporting the proposition that glucose would appear to be a suitable reference for sugary foods. In essence, glucose may be an appropriate reference for sugary foods but not for starchy foods. The opposite may also be true, that a starchy food would be an inappropriate reference for testing a sugary food. The GI of sucrose tended to be higher in the European group (88) than in the Chinese group (78) when rice was used as the reference (p = 0.19).

Interestingly, the rank order of the rice varieties we tested were largely maintained regardless of the reference food (Table 2). If GI were simply used to rank foods then the choice of reference may be of little concern. However, GI has moved on such that classifications (low, medium and high) and absolute numbers of GI now appear on food labels and in the public domain [21]. For starchy foods tested with glucose as the reference, the applicability of those classifications and values to individuals is questionable and it would be unrealistic, and contrary to the GI concept, to test and label the GI of a food specific to particular subgroups within a population. It would appear that the use of a starchy reference when testing starchy food would avoid this problem, although at present Standards Australia specify glucose as the reference [22]. The International Organization for Standards is more flexible and endorses the use of white bread or other reference foods [23].

The choice of reference, if any, may relate to the purpose of conducting the tests. For example, if we were simply interested in recommending one type of rice over another based on glycemic response then conclusions could be reached by inspection of the AUCs directly (Figure 1) without the need of a ‘reference’. This is really only valid if the same people are testing all of the foods. On the other hand, if the purpose was to make numerical comparisons among studies in which different groups of participants had been involved, then for comparability it would be desirable to settle on a standard reference for the testing of starchy foods. White bread has a history of use and although the composition may differ somewhat from location to location, in an international comparison the GI values obtained by different testing centers for white bread obtained locally were no more variable than for other starchy foods that had been provided from a single source [14]. A limitation of our conclusion that starchy foods require a starchy reference is the small number of foods tested by ourselves (five rice varieties and two breakfast cereals) and others (white bread) [8] and corn starch [15]. There is also a limited number of comparison groups (Chinese vs European; younger vs older; Caucasian vs non-Caucasian; higher vs lower salivary alpha-amylase excreters). A dependence of GI on a particular reference food may be variable among groups, for example between other ethnic groups or groups with a different age separation, thus limiting the generalizability of our findings. It would be of interest to test whether converting from one starchy reference (eg: rice) to another starchy reference (eg: white bread) would be independent of participant characteristics.

## Conclusions

For GI to be generalizable, glucose may be an appropriate reference for sugary foods but not necessarily so for starchy foods. This is hardly ideal because GI was principally devised to pertain to nutritionally appropriate starchy foods, rather than to sugary or fatty foods, on the basis of glycemic response [24].

## Abbreviations

GI: Glycaemic Index; iAUC: incremental Area Under the blood glucose Curve; BMI: Body Mass Index; CI: Confidence Interval.

## Competing interests

The authors declare that they have no competing interests.

## Authors’ contributions

BV designed the younger/older study, and MK, JM and BV designed the Chinese/European study. BV and JM wrote the draft manuscript and MK edited the script. All authors read and approved the final manuscript.
